# Action and therapeutic targets of folliculin interacting protein 1: a novel signaling mechanism in redox regulation

**DOI:** 10.3389/fcell.2025.1523489

**Published:** 2025-03-12

**Authors:** Qingzhi Ran, Aoshuang Li, Bo Yao, Chunrong Xiang, Chunyi Qu, Yongkang Zhang, Xuanhui He, Hengwen Chen

**Affiliations:** ^1^ Guang’anmen Hospital, China Academy of Traditional Chinese Medicine, Beijing, China; ^2^ Dongzhimen Hospital, Beijing University of Traditional Chinese Medicine, Beijing, China; ^3^ School of Chinese Medicine, Hong Kong Baptist University, Hong Kong, China; ^4^ Innovation Research Institute of Traditional Chinese Medicine, Shanghai University of Traditional Chinese Medicine, Shanghai, China; ^5^ Diagnosis and Treatment Center of Vascular Disease, Shanghai TCM-Integrated Hospital, Shanghai University of Traditional Chinese Medicine, Shanghai, China

**Keywords:** folliculin interacting protein 1, mitochondria, glucose sensing, autophagy, reductive stress, muscle fiber contraction, intracellular metabolism

## Abstract

Rapid activation of adenosine monophosphate-activated protein kinase (AMPK) induces phosphorylation of mitochondrial-associated proteins, a process by which phosphate groups are added to regulate mitochondrial function, thereby modulating mitochondrial energy metabolism, triggering an acute metabolic response, and sustaining metabolic adaptation through transcriptional regulation. AMPK directly phosphorylates folliculin interacting protein 1 (FNIP1), leading to the nuclear translocation of transcription factor EB (TFEB) in response to mitochondrial functions. While mitochondrial function is tightly linked to finely-tuned energy-sensing mobility, FNIP1 plays critical roles in glucose transport and sensing, mitochondrial autophagy, cellular stress response, and muscle fiber contraction. Consequently, FNIP1 emerges as a promising novel target for addressing aberrant mitochondrial energy metabolism. Recent evidence indicates that FNIP1 is implicated in mitochondrial biology through various pathways, including AMPK, mTOR, and ubiquitination, which regulate mitochondrial autophagy, oxidative stress responses, and skeletal muscle contraction. Nonetheless, there is a dearth of literature discussing the physiological mechanism of action of FNIP1 as a novel therapeutic target. This review outlines how FNIP1 regulates metabolic-related signaling pathways and enzyme activities, such as modulating mitochondrial energy metabolism, catalytic activity of metabolic enzymes, and the homeostasis of metabolic products, thereby controlling cellular function and fate in different contexts. Our focus will be on elucidating how these metabolite-mediated signaling pathways regulate physiological processes and inflammatory diseases.

## 1 Introduction

The ability to adapt to chronic nutritional deficiencies is a fundamental characteristic of all organisms’ survival. Eukaryotic cells contain highly conserved signaling pathways, which are rapidly activated when intracellular ATP levels decrease, most commonly due to a reduction in oxygen or glucose concentration that disrupts mitochondrial ATP function or in response to mitochondrial toxins or metabolic products that directly interfere with oxidative phosphorylation (OXPHOS), such as reactive oxygen species (ROS) and cytochrome C (Carlisle et al., 2016; Wu et al., 2020). Adenosine monophosphate (AMP)-activated protein kinase (AMPK) is fully phosphorylated and becomes a substrate in OXPHOS inhibition, capable of regulating lipid metabolism, glucose metabolism, mitochondrial autophagy, and mechanistic target of rapamycin complex 1 (mTORC1) signaling (Puertollano et al., 2018; Roczniak-Ferguson et al., 2012; Settembre et al., 2012; Vega-Rubin-de-Celis et al., 2017). Prolonged ATP energy stress alters transcriptional changes in gene expression programs controlling various metabolic processes (Young et al., 2016). Transcription factor EB (TFEB) and related TFE3 are activated in response to nutritional deprivation and energy stress, both inhibited by mTORC1 signaling and activated by AMPK signalin (Petit et al., 2013; Tsun et al., 2013). mTORC1 directly phosphorylates TFEB at Ser 122, Ser 142, and Ser 211, sequestration in the cytoplasm. Amino acids (AA) promote the generation of guanosine triphosphate (GTP) through the folliculin (FLCN) and FLCN interacting protein 1 (FNIP1) complex, activating the GTPase-activating protein (GAP) complex. This complex regulates the activity of small GTPases, further modulating the function of mTORC1 and affecting its ability to phosphorylate the transcription factor TFEB. TFEB, as a key transcription factor, regulates autophagy, lysosomal function, and metabolic homeostasis, playing a critical role in cellular energy metabolism, waste clearance, and other processes (Lawrence et al., 2019; Shen et al., 2019). The FLCN-FNIP1 complex determines the GTP loading of GTPase RagC, leading to the release of TFEB and TFE3 from the lysosome and nuclear translocation away from mTORC1 (Settembre et al., 2011). The FLCN/FNIP1 complex regulates the GTP loading of the GTPase RagC, promoting its activation, which drives mTORC1 translocation to the lysosome and activates its function. Furthermore, FLCN/FNIP1 regulates the RagGTPase complex, promoting the release of transcription factors TFEB and TFE3 from the lysosome and their translocation to the nucleus, thereby relieving mTORC1’s inhibition on them and regulating the expression of autophagy and metabolic genes (Tsun et al., 2013). FLCN/FNIP1 also works in concert with AMPK to respond to changes in the cell’s energy status, regulating mTORC1 activity and subsequently influencing cellular metabolic adaptive responses, such as lipid metabolism and glucose metabolism (Puertollano et al., 2018; Roczniak-Ferguson et al., 2012; Settembre et al., 2012; Vega-Rubin-de-Celis et al., 2017). Through these mechanisms, FLCN/FNIP1 plays a crucial role in cellular metabolic homeostasis, energy balance, and stress responses, ensuring that the cell can adaptively regulate according to changes in external nutrient and energy supply (Petit et al., 2013). In the nucleus, TFEB and TFE3 simultaneously bind to the DNA binding elements known as the Coordinated Lysosomal Expression and Regulation (CLEAR) motif (Perera et al., 2019). Under physiological conditions, TFEB and TFE3 remain inactive, but during specific cellular stress, they translocate to the nucleus to promote lysosomal biogenesis and possibly induce autophagy (Collodet et al., 2019). AMPK is essential for the translocation of TFEB to the nucleus during energy stress. Nazma Malik’s 2023 article published in Science confirmed that FNIP1, as a downstream protein dependent on the AMPK signaling pathway, can dictate the translocation of TFEB and TFE3 to the nucleus, facilitating the transcription of CLEAR genes (Malik et al., 2023).

**FIGURE 1:**
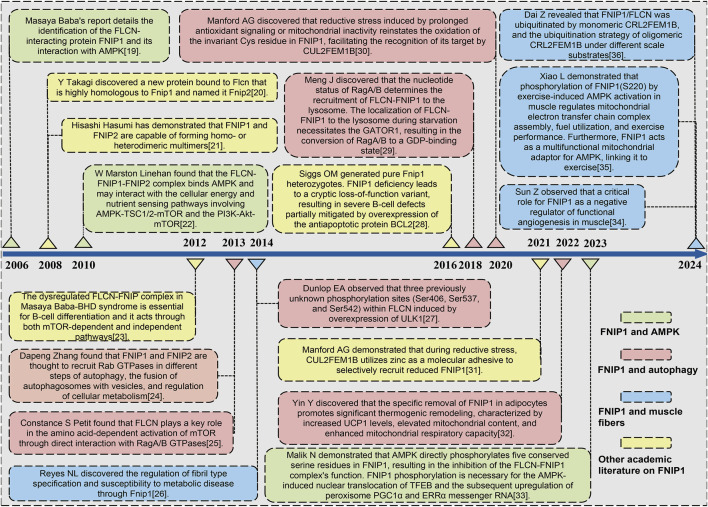
Timeline of key milestones in the development of FNIP1 signaling. Abbreviations: FLCN, Folliculin; FNIP1, Folliculin Interacting Protein 1; AMPK, Adenosine 5′-monophosphate (AMP)-activated protein kinase; mTOR, Mammalian target of rapamycin. TSC1, Tuberous Sclerosis Complex 1; PI3K, Phosphatidylinositide 3-kinase; AKT1, AKT serine/threonine kinase 1; GATOR1, GAP activity toward Rags 1; GDP, Guanosine diphosphate; PGC1α Peroxisome proliferators-activated receptor γ coactivator l alpha; ERRα, Estrogen Receptor alpha.

The precise mechanism through which AMPK activates the transcriptional program for mitochondrial biogenesis via FNIP1 has been elucidated. Nazma Malik’s team conducted a time-course analysis of the transcriptional response to mitochondrial OXPHOS inhibitors, revealing a temporal cascade of organelle biogenesis that is genetically dependent on AMPK (Hung et al., 2021). It has been determined that FNIP1, as a direct AMPK substrate, plays a crucial role where its phosphorylation is essential for the activation and nuclear translocation of TFEB. This, in turn, leads to the production of Peroxisome Proliferator-Activated Receptor Gamma, Coactivator 1 Alpha (PGC-1α) and Estrogen-Related Receptor α (ERRα) mRNA, triggering lysosomal biogenesis responses and thus affecting mitochondrial biological processes (Cantó and Auwerx, 2009). FNIP1 acts as a direct substrate of AMPK through the AMPK signaling pathway, and is phosphorylated upon AMPK activation, thereby regulating the transcriptional activity of TFEB (Tsun et al., 2013). Upon AMPK activation, phosphorylation of FNIP1 promotes the translocation of TFEB from the lysosome to the nucleus, activating the transcription of genes related to organelle biogenesis, including Peroxisome Proliferator-Activated Receptor Gamma Coactivator 1 Alpha (PGC-1α) and Estrogen-Related Receptor Alpha (ERRα) (Shen et al., 2019). PGC-1α, as a key regulator of mitochondrial biogenesis, promotes mitochondrial generation and functional recovery, while ERRα regulates mitochondrial metabolic responses (Collodet et al., 2019). Through this process, FNIP1 helps cells maintain energy homeostasis and metabolic adaptation by promoting mitochondrial biogenesis and functional regulation under stress conditions, thereby enhancing the cell’s ability to respond to energy depletion and oxidative stress. We have compiled a timeline of key milestone developments in mitochondrial diseases, cardiovascular diseases, and skeletal muscle disorders where Fnip1 signaling has been implicated, as shown in Figure 1 (Baba et al., 2006; Baba et al., 2012; Dai et al., 2024; Dunlop et al., 2014; Hasumi et al., 2008; Linehan, Srinivasan and Schmidt, 2010; Manford et al., 2021; Manford et al., 2020; Meng and Ferguson, 2018; Petit et al., 2013; Reyes et al., 2015; Rezvan et al., 2024; Siggs et al., 2016; Sun et al., 2023; Takagi et al., 2008; Xiao et al., 2024; Yin et al., 2022; Zhang et al., 2012). Given the novelty and complex pathophysiological mechanisms of FNIP1, this review for the first time retrospectively summarizes the research trajectory and milestones of FNIP1, attempts to summarize the physiological mechanisms of FNIP1 in mitochondria and its pathological mechanisms in various diseases, laying the foundation for understanding the multilevel regulatory signaling pathways and mechanisms of FNIP1. This provides a reference for future development of FNIP1-targeted drug therapies for mitochondrial diseases and improving patients prognosis with reduced stress, inflammatory response, skeletal muscle damage, et.

## 2 Molecular characterization of FNIP1

**FIGURE 2:**
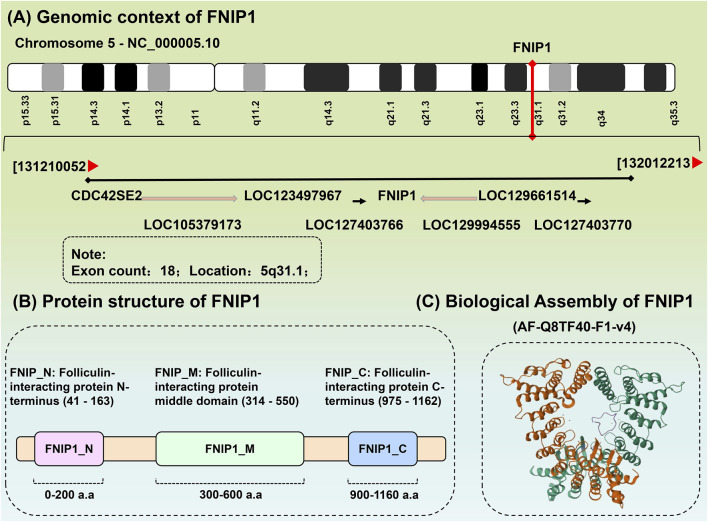
Biochemical structure of FNIP1. **(A)** The genomic background of FNIP1. **(B)** Protein structure of FNIP1. **(C)** Biological assembly of FNIP1.FNIP1, Folliculin Interacting Protein 1. (1) Gene annotation: The FNIP1 gene is located at the center of the diagram (marked in red), with an arrow indicating its direction, representing its transcription direction. Other genes related to FNIP1, such as CDC42SE2, LOC123497967, and LOC129661514, are listed around it, with their transcription directions indicated by arrows. (2) Chromosomal location: The position of the FNIP1 gene is labeled as 5q31.1, indicating that it is located at position 31.1 on the q arm (long arm) of chromosome 5. Its genomic location in the diagram is marked from 131210052 to 132012213, representing the chromosomal region where the FNIP1 gene is found. (3) Number of exons: The annotation in the diagram indicates that the FNIP1 gene has 18 exons. Exons are the coding regions of the gene that, after transcription, are converted into mRNA and involved in protein synthesis. (4) Relative position and relationship of genes: The diagram uses arrows to indicate the relative positions of other genes, showing their locations and transcription directions near the FNIP1 gene. These genes may be related to the function or regulation of FNIP1, such as CDC42SE2 and LOC123497967. (5) Arrows indicate the transcription direction of genes: The direction of each arrow represents the transcription direction of the gene, which is the direction of mRNA synthesis. The direction of the arrow indicates that the transcription of the gene occurs from the 5′ end to the 3′ end, which is the general rule of gene transcription. These arrows are primarily unidirectional, indicating the transcription direction of the gene. In some cases, the arrow may be reversed (e.g., from right to left), indicating that the corresponding gene is transcribed in the reverse direction.

FNIP1 is ubiquitously distributed in 27 different tissues including the heart, testes, and thyroid, with RNA sequence analysis of FNIP1 across these 27 human tissues presented in Table 1. The FNIP1 gene encodes a protein that interacts with the tumor suppressor protein folliculin and AMPK, participating in the regulation of cell metabolism and nutrient sensing through the mTOR pathway (Manford et al., 2020). This gene has a closely related paralog that encodes a protein with similar binding activity. These two related proteins bind to the molecular chaperone Heat Shock Protein-90 (Hsp90) and negatively regulate its ATPase activity, promoting its interaction with folliculin. The genomic context of FNIP1 is shown in Figure 2A, in the context of Chromosome 5 - NC_000005.10. (Exon count:18; Location:5q31.1). Listing from CDC42SE2 to ACSL6-AS1. The protein structure of FNIP1 is depicted in Figure 2B (Zhang et al., 2012), FNIP_N: Folliculin-interacting protein N-terminus (41–163), FNIP_M: Folliculin-interacting protein middle domain (314–550), FNIP_C: Folliculin-interacting protein C-terminus (975–1,162). And the biological assembly of FNIP1 is illustrated in Figure 2C (Petit et al., 2013; Zhang et al., 2012). We used AlphaFold (https://alphafold.ebi.ac.uk/) for homology modeling. This tool predicts the three-dimensional structure of FNIP1 by aligning it with known homologous protein structures. The identifier for FNIP1 on the AlphaFold website is AF-Q8TF40-F1-v4. Furthermore, Hisashi Hasumi identified a new FLCN-interacting protein, FNIP2, which is homologous to FNIP1 across species and has been shown to form polymers independent of FLCN expression, functioning cooperatively with FLCN. Tissue-specific expression patterns suggest potential tissue specificity in normal cellular signal transduction for FNIP1 and FNIP2. The sequence alignment of FNIP1 and FNIP2, along with key information such as the homology percentage, similarity, and differences between the isoforms, can be found in Supplementary Figure 1. The differences in amino acid sequence homology, domain differences, tissue distribution, and cellular functions between FNIP1 and FNIP2 are summarized in Table 2 (Manford et al., 2021; Yin et al., 2022).

**TABLE 1:** RNA sequence analysis of FNIP1 in 27 human tissues.

Sample	BioSample	RPKM	Count
adrenal	3 samples	5.058 ± 0.36	382,622
appendix	3 samples	4.32 ± 1.227	282,768
bone marrow	4 samples	5.298 ± 2.389	978,646
brain	3 samples	6.144 ± 0.914	547,990
colon	5 samples	4.572 ± 0.577	875,515
duodenum	2 samples	4.332 ± 0.051	199,821
endometrium	3 samples	5.601 ± 0.399	473,138
esophagus	3 samples	3.568 ± 0.844	450,553
fat	3 samples	5.46 ± 0.901	422,333
gall bladder	3 samples	5.501 ± 0.974	673,783
heart	4 samples	3.42 ± 0.393	588,982
kidney	4 samples	4.27 ± 0.501	392,849
liver	3 samples	3.655 ± 0.319	324,209
lung	5 samples	3.929 ± 0.835	540,926
lymph node	5 samples	3.319 ± 0.696	712,829
ovary	2 samples	6.66 ± 0.061	656,164
pancreas	2 samples	0.854 ± 0.015	83,310
placenta	4 samples	4.132 ± 1.243	710,143
prostate	4 samples	5.136 ± 0.254	504,367
salivary gland	3 samples	0.965 ± 0.062	153,614
skin	3 samples	3.18 ± 0.884	425,579
small intestine	4 samples	3.716 ± 0.586	402,755
spleen	4 samples	3.699 ± 0.589	523,057
stomach	3 samples	3.868 ± 0.756	408,452
testis	7 samples	9.118 ± 0.933	2,968,022
thyroid	4 samples	7.446 ± 2.167	1,371,762
urinary bladder	2 samples	4.294 ± 0.157	348,296

Note: RPKM, reads per kilobase of exon model per million mapped reads.

**TABLE 2:** Comparison of amino acid sequence homology, domain differences, tissue distribution, and cellular functions between FNIP1 and FNIP2.

Category	FNIP1	FNIP2
Amino Acid Sequence Homology	60%–70% sequence homology, highly conserved Folliculin Interacting Domain, differences in GTPase binding and lysine-rich motifs	60%–70% sequence homology, similar Folliculin Interacting Domain, distinct regions affecting neural and muscle regulation
Domain Differences	Key role in autophagy regulation, especially in kidney and cancer cells	Plays a more significant role in the nervous system and muscle cells, especially in energy metabolism
Tissue Distribution	Kidneys (tubular cells and renal epithelial cells), tumor cells (renal cell carcinoma and lung cancer), lungs, and brain	Brain (neurons and glial cells), heart, skeletal muscle
Cell Function	Regulates energy metabolism via AMPK, involved in autophagy and cell proliferation, tumor suppression Role in metabolic regulation, cellular response to stress, regulation of autophagy, and nutrient sensing	Regulates energy metabolism, particularly in neurons and muscle cells, affecting cell survival and function Essential in muscle and neuron metabolic regulation, adjusts energy balance in response to activity

Note: FNIP1, Folliculin Interacting Protein 1; FNIP2, Folliculin Interacting Protein 2.

## 3 AMPK and FNIP1: interconnected regulators of cellular energy metabolism and mTOR signaling pathways in health and disease

AMPK is a cellular energy sensor and a crucial kinase regulating energy homeostasis, serving as one of the central regulators of cell and organism metabolism in eukaryotes. It is responsible for monitoring cellular energy input and output to maintain the smooth operation of cellular physiological activities (Rezvan et al., 2024). AMPK also plays a key role in multiple signaling pathways. It is activated under conditions of energy deficiency, and the classical AMPK signaling pathway is associated with increased AMP/ATP and ADP/ATP ratios (Sun et al., 2023). Activation of AMPK promotes ATP production through anabolic pathways and inhibits energy expenditure to restore energy balance. Additionally, AMPK, as a key regulatory factor in cellular energy sensing, maintains energy balance through various mechanisms when cellular energy is insufficient (Vega-Rubin-de-Celis et al., 2017). First, AMPK promotes ATP synthesis by activating catabolic pathways (such as glycogen breakdown and fatty acid oxidation), alleviating the intracellular environmental disturbance caused by cellular energy metabolism imbalance. Meanwhile, AMPK inhibits anabolic pathways (such as fatty acid synthesis and protein synthesis), reducing ATP consumption and optimizing the efficiency of cellular energy utilization (Xiao et al., 2024). In addition, AMPK regulates carbohydrate and lipid metabolism by activating the glucose transporter GLUT4 and enhancing the glycolytic pathway, as well as activating key enzymes of fatty acid oxidation (such as carnitine palmitoyltransferase 1 (CPT1)). AMPK also promotes mitochondrial repair and biogenesis by regulating genes related to mitochondrial biogenesis, such as PGC-1α (Carlisle et al., 2016; Collodet et al., 2019).

**FIGURE 3:**
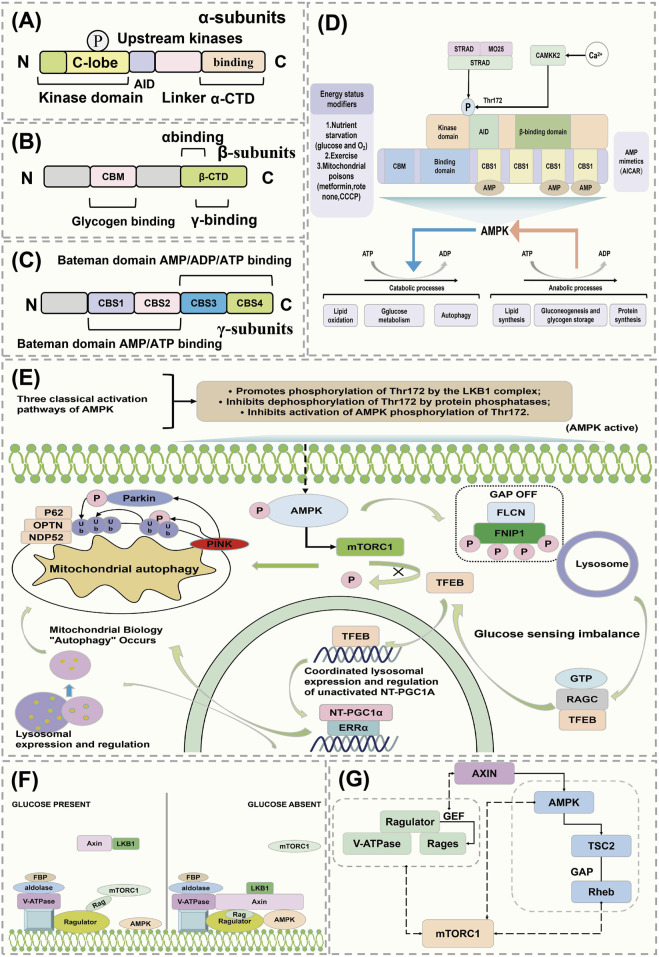
AMPK regulates the mechanism of action of FNIP1. **(A–C)** AMPK conventional kinase structure. **(D)** AMPK maintains energy balance. **(E)** Mechanism of action of AMPK activation of FNIP1. **(F)** Activation pathway of AMPK. **(G)** Mutual regulation of mTOR and AMPK. AID, Activation-induced cytidine deaminase; STRAD, STE20-related kinase adaptor protein; MO25, MO25 alpha/beta (MO25 forms a complex with LKB1 and STRAD proteins involved in the regulation of cell polarity and metabolism); CAMKK2, Calcium/calmodulin-dependent protein kinase kinase 2; CBM, Carbohydrate-binding modules; CBS1, Cystathionine beta-synthase 1; PGC1α, peroxisome proliferators-activated receptor γ coactivator l alpha; OPTN, Optineurin; FBP, Fatty acid-binding protein; LKB1, Liver kinase B1; AXIN, Axis Inhibition Protein 1; TSC2, Tuberous Sclerosis Complex.

The conventional kinase domain of AMPK is located at the N-terminus of the α-subunit, followed by an autoinhibitory domain (AID). Following the AID is an extended linker peptide (highlighted in red in Figure 3A), which connects the AID to the C-terminal domain of the α-subunit (α-CTD) (Figure 3A) (Parmar et al., 2022). The β-subunit of AMPK contains a carbohydrate binding module (CBM) that may be involved in binding to targets such as glycogen synthase, although this function is not yet confirmed. The C-terminal domain of the β-subunit (β-CTD) interacts with the α-CTD and the γ-subunit, forming the core of the complex (Figure 3B) (Moreno-Corona et al., 2023). The γ-subunit of AMPK contains four tandem repeats of the CBS motif (CBS1-CBS4). These tandem repeats occur in a few other proteins, such as cystathionine β-synthase, and typically assemble as two repeats to form a Bateman domain, with ligand-binding sites in the crevices between the repeats (Hasumi et al., 2015). The CBS core contains four potential ligand-binding sites (Figure 3C). Upstream kinase CAMKK2 is activated by intracellular calcium ions, and the heterotrimeric complex of LKB1 with STRAD and MO25 triggers the mechanistic activation of AMPK (Zhang L. et al., 2023). Activators of AMPK include oxygen stress, glucose starvation, exercise, and mitochondrial toxins. The function of AMPK activation is to promote catabolic metabolism and reduce anabolic processes, decrease ATP consumption, increase ATP synthesis, and maintain energy balance (Figure 3D) (Wang X. et al., 2023).

FNIP1 is a conserved substrate of AMPK that controls the phosphorylation status and localization of TFEB. FNIP1 is an established interacting partner of FLCN and has been reported to co-immunoprecipitate with AMPK (Cai et al., 2024), although the specific details of this regulation by AMPK are still unclear. The FNIP1-FLCN complex acts as an amino acid sensor affecting the mTORC1 signaling pathway, with amino acids playing a role in controlling the activation of TFEB (41). Studies have found that AMPK can regulate FNIP1 to control TFEB independently of amino acids (Herzig and Shaw, 2018). The phosphorylation of FNIP1 by AMPK-dependent mechanisms controls the interaction between mTORC1 and TFEB/TFE3. Phosphorylation of FNIP1 by AMPK inhibits the GAP activity of the FNIP1-FLCN complex, controlling the transcription factor TFE via RagC (Figure 3E) (Carling, 2017; Herzig and Shaw, 2018; Wang X. et al., 2023).

Recent research has revealed that AMPK can sense cellular energy status and also detect glucose availability independently of adenylate changes, through an aldolase mechanism (Trefts and Shaw, 2021). AMPK and mTOR interact, where mTOR can activate AMPK through non-classical pathways, while also being subject to effective regulation by AMPK (Kim et al., 2016). Under high glucose conditions, aldolase is occupied by fructose-1,6-bisphosphate (FBP), and the Rag activity regulator converts RagA/B to the GTP-bound form, recruiting mTORC1 to the lysosome where mTORC1 may be activated under suitable conditions (GTP-bound forms of RagC/D, GTP-bound form of Rheb) (González et al., 2020). Under low glucose conditions, the absence of FBP in aldolase leads to changes in its interaction with the V-ATPase, causing dissociation of mTORC1 and forming a ‘supercomplex’ that includes V-ATPase, regulator, AXIN, LKB1, and AMPK (based on the AXIN-AMPK activation complex), thereby triggering phosphorylation of AMPK on Thr172, thus activating AMPK (Figure 3F) (Chen et al., 2024; Freemantle and Hardie, 2023). mTORC1 activity is regulated by two parallel mechanisms: under glucose depletion, the regulator’s GEF activity towards RAGs is inhibited, converting RAG to the GDP-bound form and inhibiting mTORC1 (Ramirez Reyes et al., 2021). Additionally, AXIN binds with the regulator, inhibiting its GEF activity towards RAGs. Activated AMPK can also phosphorylate TSC2 and activate its GAP activity to inactivate Rheb, or directly phosphorylate key components of mTORC1 such as Raptor, leading to inhibition of mTORC1 (Figure 3G) (Paquette et al., 2021; Ross et al., 2023).

## 4 FNIP1 as a central node in cellular homeostasis: integrating mitophagy, DNA repair, and lysosomal function through AMPK signaling

**FIGURE 4:**
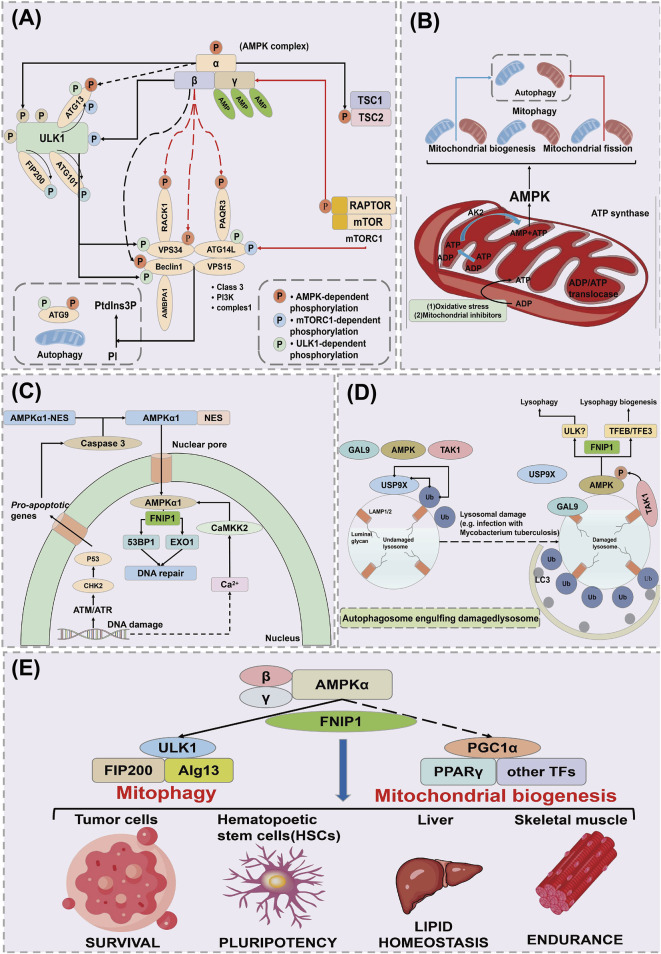
AMPK regulates the mechanism of action of FNIP1. **(A)** Mechanisms of cellular autophagy. **(B)** AMPK acts on mitochondrial autophagy through FNIP1. **(C)** AMPK acts on DNA damage and repair through FNIP1. **(D)** AMPK acts through FNIP1 for lysosomal injury. **(E)** AMPK regulates autophagy through FNIP1 to affect various physiological functions. ULK1, Unc-51 like autophagy activating kinase 1; ATG13, Autophagy Related 13; FIP200, FAK family kinase-interacting protein of 200 kDa; RACK1, Receptor of activated protein kinase C1; VPS34, Vacuolar Protein Sorting 34; ATG14L, Autophagy-related protein 14-like; AMBPA1, Autophagy and beclin 1 regulator 1 gene; ATG9, Autophagy-related protein 9; EXO1, Exonuclease 1; CHK2, Checkpoint kinase2; GAL9, Growth arrest-specific protein 9; TAK1, Transforming growth factor beta-activated kinase 1; USP9X, Ubiquitin Specific Peptidase 9X-linked.

Autophagy is a process by which cells maintain homeostasis, clear waste, and reconstruct cellular structures by self-ingesting and degrading damaged or unnecessary materials within the cell (such as damaged organelles, excess proteins, and pathogens) under both internal and external environmental stress (Russell and Hardie, 2020). Autophagy plays an important role in cellular physiological processes and also serves a protective function in helping cells respond to environmental challenges such as starvation, oxidative stress, and infection. Autophagy is mainly regulated through essential genes-ATG. During the process of autophagy, ULK1 is phosphorylated and activates a PI3K complex composed of VPS34, VPS15, ATG14L, and Beclin1 (MacDonald et al., 2018). This complex generates phosphatidylinositol 3-phosphate (PtdIns3P) on nascent autophagosome membranes, forming an autophagic membrane that includes ATG9 (Zhang et al., 2017). ATG9 is the only transmembrane ATG protein, which is activated by the action of ATG4, ATG7, and ATG3 (LC3I), producing LC3II (Zhang et al., 2014). LC3II serves as a marker for autophagy to visualize autophagosomes and quantify autophagy in cells, and it can bind to autophagy receptors such as SQSTM1, NDP52, NBR1, and OPTN (Chen et al., 2017). Subsequently, autophagosomes mature and fuse with lysosomes in a process mediated by a complex composed of VPS34, VPS15, Beclin1, and UVRAG, inducing lysosomal enzymes to degrade targeted cells (Figure 4A) (Kwiatkowski and Manning, 2005).

### 4.1 FNIP1 and mitophagy

AMPK plays a crucial role in sensing and responding to mitochondrial damage by directly phosphorylating FNIP1, leading to the inhibition of the FLCN binding GAP activity of FNIP1 (Avruch et al., 2006). This results in the accumulation of Rag C in its GTP-bound form, causing the dissociation of Rag C, the mTORC1, and the transcription factor EB (TFEB) from the lysosome (Terešak et al., 2022). AMPK plays a crucial role in sensing and responding to mitochondrial damage. AMPK directly phosphorylates FNIP1, inhibiting the GAP activity of FLCN, leading to the accumulation of RagC in its GTP-bound form. The GTP-bound form of RagC promotes its activation and forms a complex with RagA, subsequently activating mTORC1, which translocates to the lysosome. In this process, mTORC1 phosphorylates TFEB, inhibiting its nuclear translocation and preventing TFEB from activating the expression of autophagy-related genes (Vega-Rubin-de-Celis et al., 2017). However, when RagC and mTORC1 are activated, mTORC1 dissociates and relieves the inhibition on TFEB, promoting TFEB’s translocation to the nucleus, where it activates the transcription of autophagy and lysosomal biogenesis genes, thereby initiating cellular autophagic and metabolic responses (Ramirez Reyes et al., 2021; Wang X. et al., 2023). Through this mechanism, RagC and mTORC1 regulate autophagic activity in cells under stress conditions, helping cells adapt to energy depletion or damaged environments. Unphosphorylated TFEB translocates to the nucleus, inducing the transcription of lysosomal or autophagy genes, while NT-PGC 1α mRNA levels increase in parallel, consistent with Estrogen-Related Receptor α (ERRα), inducing changes in mitochondrial structure such as enhancing Ca^2+^ exchange between the endoplasmic reticulum and mitochondria. This leads to mitochondrial Ca^2+^ overload, excessive opening of the mitochondrial permeability transition pore (mPTP), and release of cytochrome C (Cyt C), causing mitochondrial dysfunction and disturbances in energy metabolism (Kim et al., 2011). Due to oxidative damage or chemical inhibitors, the F1 ATP synthase located on the cristae surface is inhibited, increasing the mitochondrial inner ADP/ATP ratio (Kumariya et al., 2021). This is conveyed through an increased ADP/ATP ratio in the intermembrane/cristae space via the ATP/ADP translocase, in turn converting ADP to AMP via the catalytic action of adenylate kinase (AK2). This reversible conversion activates AMPK, thereby promoting mitochondrial biogenesis, fission, and mitophagy to maintain mitochondrial homeostasis (Figure 4B) (Sun et al., 2021).

### 4.2 FNIP1 and DNA damage and repair

DNA damage leads to an increase in nuclear Ca^2+^, potentially due to its release from the nuclear envelope, although its exact source remains unclear. Concurrently, DNA damage may trigger apoptosis through the activation of the DNA damage-sensitive kinases ATM/ATR and CHK2 downstream of p53 (Hao et al., 2022). This results in the activation of cysteine-aspartic acid protease 3 (caspase 3), which cleaves the nuclear export signal (NES) of AMPK α1, causing its translocation from the cytoplasm to the nucleus (Filali-Mouncef et al., 2022). Within the nucleus, the AMPK complex containing α1 is phosphorylated and activated by Ca^2+^/calmodulin-dependent protein kinase kinase 2 (CaMKK2), triggered by the elevation of Ca^2+^ (Heo et al., 2018). AMPK phosphorylates and inhibits the exonuclease EXO1 and the p53 binding protein 1 (53BP1), assisting in DNA damage repair (Figure 4C) (Tudorica et al., 2024).

### 4.3 FNIP1 and lysosomal damage

In undamaged lysosomes, the deubiquitinating enzyme USP9X removes ubiquitin clusters from the lysosomal surface (Yamano et al., 2024). Lysosomal damage exposes the lumenal glycosides on LAMP1/LAMP2, recruiting the cytosolic lectin galectin-9 (GAL9) (Marchesan et al., 2024). This, in turn, recruits AMPK and Transforming Growth Factor-Beta Activated Kinase 1 (TAK1) to the lysosomal surface, displacing USP9X. The absence of USP9X enhances the formation of ubiquitin clusters on the lysosomal surface, thus triggering autophagosome engulfment, and also promotes the formation of K63-linked polyubiquitin chains on the TAK1 complex, leading to its activation and subsequent activation of AMPK (Jia et al., 2022; Nguyen et al., 2023; Shariq et al., 2024). AMPK promotes lysophagy, possibly involving the phosphorylation of ULK1, while simultaneously activating TFEB/TFE3 to stimulate the biogenesis of new lysosomal components (Figure 4D) (Arhzaouy et al., 2019; Nakamura et al., 2021). After AMPK activation, it phosphorylates TSC2 and RAPTOR, leading to the downregulation of the mTOR complex 1 (mTORC1), which consists of mTOR, RAPTOR, mLST8, DEPTOR, and PRAS40 (Cheng et al., 2018; Wang et al., 2024). AMPK activation also phosphorylates ULK1, enhancing its activity and initiating the autophagic process (Jolly et al., 2009). Activated AMPK stimulates mitophagy through ULK1, while simultaneously promoting new mitochondrial production via transcriptional regulation through PGC-1α (Premarathne et al., 2017). This dual regulation process of AMPK can replace defective mitochondria with new functional ones, achieving a “cleansing effect” of the mitochondria (Figure 4E) (Jaiswal et al., 2021).

## 5 FNIP1 and regulation of the reductive stress response

Redox stress refers to the stress response caused by the imbalance of oxidation-reduction within the intracellular and extracellular environment. This imbalance leads to the generation of large amounts of ROS, subsequently causing lipid peroxidation of cell membranes, protein oxidation, and DNA damage (Liubomirski et al., 2019). While oxidative phosphorylation can efficiently produce ATP, it also generates ROS as a metabolic byproduct. When ROS consumption is below its physiological level (The consumption of ROS within the cell is lower than what is required for normal physiological conditions) (Reyes et al., 2015), this phenomenon is referred to as reductive stress, which is an imbalance in redox state due to either an excessive reduction capacity or an abundance of high-reduction potential substances within the organism (Izrailit et al., 2017). Redox stress plays a crucial role in the development of various diseases, such as cardiovascular diseases, neurodegenerative diseases, and inflammatory diseases. Oxidative Stress (OS) results from the accumulation of ROS, leading to a disturbance in the cellular redox state. The steady-state level of ROS is important for cell physiology and signaling, but excessive ROS can induce reductive stress, ranging from the destruction of biomolecules to apoptosis (Inoki et al., 2002). Additionally, oxidative stress can impair the function of redox-sensitive organelles, including mitochondria and the endoplasmic reticulum (ER).

Andrew G Manford in Cell demonstrated that cells mitigate reductive stress by ubiquitination and degradation of the mitochondrial gatekeeper FNIP1, with CUL2FEM1B relying on zinc as a molecular glue to selectively recruit reduced FNIP1 during reductive stress (Huang and Manning, 2009; Inoki et al., 2002; Izrailit et al., 2017; Liubomirski et al., 2019). FNIP1 ubiquitination is gated by the pseudo-substrate inhibitors of the BEX family, preventing premature degradation of FNIP1 to protect cells from unnecessary ROS accumulation. Functional mutations in FEM1B and deficiencies in BEX result in similar developmental syndromes, suggesting that zinc-dependent reductive stress responses must be tightly regulated to maintain cellular and organismal homeostasis (PMID: 34562363) (Hu et al., 2021). Wenqian Zhang found that FNIP1 improves oxidative stress and rescues impaired angiogenesis in HUVECs under high glucose conditions (Wang and Jia, 2023). Zhang developed a novel glucose-responsive HA-PBA-FA/EN106 hydrogel for localized drug delivery and regulation of FNIP1 at diabetic wound sites. Due to the dynamic boronate ester bonds between hyaluronic acid (HA) and phenylboronic acid (PBA), the hydrogel enables glucose-responsive drug release. The release of the FEM1b-FNIP1 axis inhibitor EN106 through the regulation of the FEM1b-FNIP1 axis mitigates oxidative stress and stimulates angiogenesis, offering a promising strategy for chronic diabetic wound healing, and also serves as proof of FNIP1 as an effector of reductive stress (Paul et al., 2023).

## 6 FNIP1 and mitochondrial maintenance in skeletal muscle metabolic homeostasis

**FIGURE 5:**
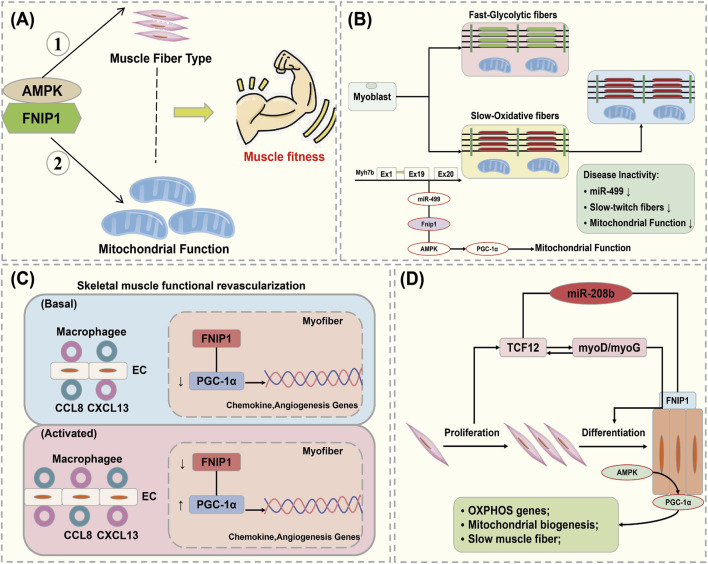
Interaction of FNIP1 with autophagy. **(A)** FNIP1 and myofiber mitochondrial function, AMPK independent; **(A)** AMPK/PGC-1 dependent. **(B)** FNIP1 signaling pathway affects muscle fiber function. **(C)** FNIP1 and blood vessels. **(D)** FNIP1 and muscle fiber homeostasis. EC, endothelial cell; CCL8, C-C motif chemokine ligand 8; CXCL13, C-X-C motif ligand 13; TCF12, Transcription Factor 12.

The maintenance of mitochondrial function is crucial for skeletal muscle metabolic homeostasis under both physiological and pathological conditions. Mitochondrial function, muscle fiber contraction, and vascular damage reconstruction all contribute to the physiological mechanisms that maintain muscle function. Xiao L discovered that FNIP1 is associated with skeletal muscle fiber type specification, function, and disease. FNIP1 is specifically expressed in the skeletal muscle of FNIP1 transgenic (FNIP1Tg) mice, and it was found that FNIP1 influences muscle mitochondrial oxidation processes through AMPK signaling (Henning et al., 2022). The action of FNIP1 on type I muscle fiber programming is independent of AMPK and its downstream target PGC-1α. This suggests that FNIP1 serves as a key factor in the coordinated regulation of mitochondrial function and muscle fiber type, which are determinants of muscle health (Figure 5A) (Gilder et al., 2013).

Exercise-induced activation of AMPK and substrate phosphorylation regulates mitochondrial metabolic capacity in skeletal muscle. Liwei Xiao found that AMPK phosphorylation of FNIP1 at serine-220 (S220) controls mitochondrial function and muscle fuel utilization during exercise. The absence of FNIP1 in skeletal muscle leads to increased mitochondrial content and enhanced metabolic capacity, thereby enhancing exercise endurance in mice, and demonstrating that muscle AMPK-induced phosphorylation of FNIP1 (S220) regulates mitochondrial electron transport chain complex assembly, fuel utilization, and exercise performance. Thus, FNIP1 is a multifunctional AMPK effector used for the mechanisms of mitochondrial adaptation to exercise capacity and endurance (Barford, 2011). When skeletal muscle adapts to physiological and pathophysiological stimuli, muscle fiber type and mitochondrial function are coordinately regulated. Preliminary studies identified pathways involved in the control of oxidative fiber contraction proteins. Jing Liu elucidated the mechanisms coupling mitochondrial function with muscle contraction mechanisms during fiber type transition, where the expression of genes encoding type I myosin proteins, Myh7/Myh7b, and their intronic miR-208b/miR-499 is parallel to mitochondrial function during fiber type transition. miR-499 drives PGC-1α-dependent mitochondrial oxidative metabolism to match the changes in slow muscle fiber composition. It was demonstrated that miT-499 directly targets FNIP1, serving as a negative regulator of the AMPK interacting protein (Wang Y. et al., 2023). This implies that inhibiting FNIP1 reactivates AMPK/PGC-1α signaling and mitochondrial function in myocytes, and identifies the miR-499/Fnip1/AMPK signaling pathway as a regulator of muscle fiber type and mitochondrial function mechanisms (Figure 5B) (Tsai et al., 2024).

The main cause of damage and apoptosis in cardiomyocytes and skeletal muscle cells is impaired blood supply, where mechanisms of functional angiogenesis protect against the damage to myocardial and skeletal muscles by preserving blood flow. Zongchao Sun’s research discovered that FNIP1 controls functional angiogenesis in skeletal muscles, reconstructing muscle blood flow during ischemia. Muscle FNIP1 expression is downregulated by exercise. Genetic overexpression of FNIP1 in muscle fibers restricts angiogenesis in mice, whereas its fiber-specific ablation significantly promotes the formation of functional blood vessels. The increase in muscle vascularization is independent of AMPK but is due to enhanced macrophage recruitment in muscle cells depleted of FNIP1 (Zhang W. et al., 2023). FNIP1 deficiency in muscle fibers induces the activation of PGC-1α and chemotactic factor gene transcription, thereby driving macrophage recruitment and muscle vascularization programs, and the absence of muscle fiber FNIP1 significantly improves blood flow recovery (Figure 5C) (Zhu et al., 2023). Embryonic and neonatal skeletal muscles grow through the proliferation and fusion of myogenic cells, while adult skeletal muscles adapt mainly by remodeling pre-existing fibers and optimizing metabolic balance. Fu L’s research found that miR-208b stimulates the transition between fast and slow fibers and oxidative metabolism programs by targeting FNIP1 rather than the TCF12 gene. Moreover, miR-208b targeting of FNIP1 activates AMPK/PGC-1α signaling and mitochondrial biogenesis. Therefore, miR-208b specifically targets TCF12 and FNIP1 to mediate skeletal muscle development and homeostasis (Figure 5D) (Sopariwala et al., 2021).

## 7 FNIP1: a multifaceted protein in tumor development and metabolic regulation

**FIGURE 6:**
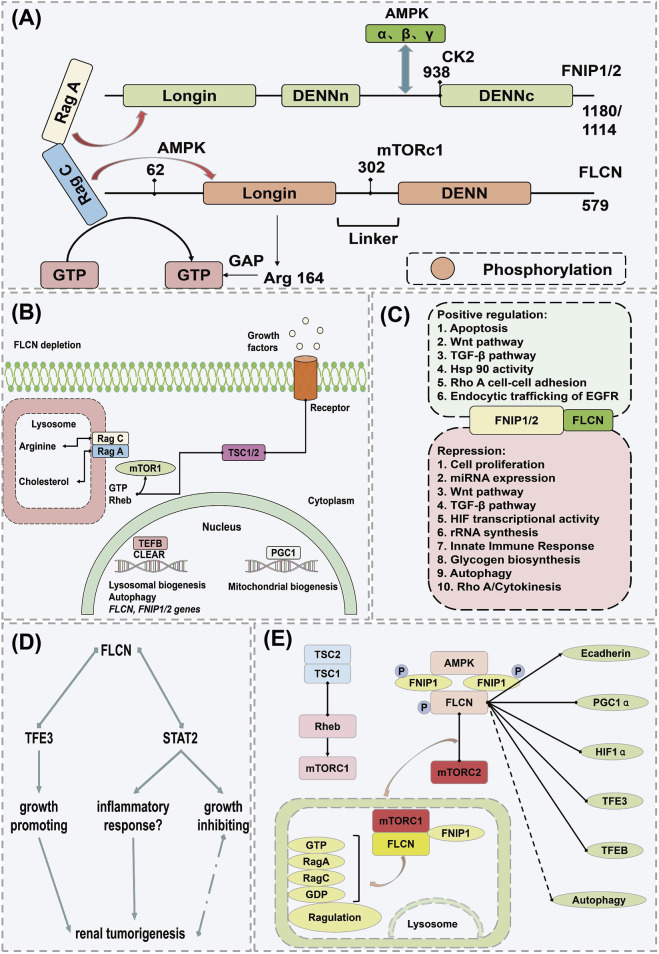
Mechanism of action of FNIP1 in causing cancer. **(A)** Structural domains of FLCN and FNIP and their interacting proteins. **(B)** Inactive state of Rag C/D under FLCN depletion. **(C)** Additional pathways and cellular processes regulated by the FLCN/FNIP complex. **(D)** Mechanistic pathway of FLCN knockout. **(E)** FLCN-related pathways potentially leading to renal tumor formation under FLCN deficiency. Arrows indicate activation, and dots represent inhibition; if both symbols are present, the interaction is either conflicting or unclear. AMPK, Adenosine 5′-monophosphate (AMP)-activated protein kinase; CAMKK2, Calcium/calmodulin-dependent protein kinase kinase 2; ERRα, Estrogen Receptor alpha; FLCN, Folliculin; FBP, Fatty acid-binding protein; FNIP1, Folliculin Interacting Protein 1; GATOR1, GAP activity toward Rags 1; GAL9, Growth arrest-specific protein 9; GDP, guanosine diphosphate; PGC1α, Peroxisome proliferators-activated receptor γ coactivator l alpha; STRAD, STE20-related kinase adaptor protein; TAK1, Transforming growth factor beta-activated kinase 1; TSC1, Tuberous Sclerosis Complex 1; TSC2, Tuberous Sclerosis Complex; TCF12, Transcription Factor 12.

FNIP1 is a protein associated with multiple biological processes and diseases, playing a particularly complex role in the development and progression of tumors (Rowe et al., 2014). FNIP1, closely linked to oncological functions, was initially identified as an interacting protein of the tumor suppressor gene Folliculin (FLCN) (Figure 6A). The Folliculin (FLCN) gene, identified in 2002, is responsible for Birt-Hogg-Dubé (BHD) syndrome, an autosomal dominant genetic disorder characterized by fibrofolliculomas, pulmonary cysts, increased spontaneous pneumothorax frequency, and bilateral multifocal renal tumors (Blenis, 2017; Hardie, 2011). Folliculin forms complexes with two proteins, FNIP1 and FNIP2, serving as functional partners of FLCN. The dual inactivation of FNIP1/2 in mouse kidneys leads to polycystic kidney enlargement akin to FLCN deficiency, highlighting their roles as tumor suppressors, as mice with FNIP1 and/or FNIP2 knockouts exhibit tumors in multiple organs (Erdogan et al., 2016). As anticipated, the regulatory role of the FLCN/FNIP complex in Rab GTPases and membrane trafficking is delineated (Figure 6B) (Lushchak et al., 2017; Pan et al., 2012). FLCN, a pleiotropic protein, participates in numerous cellular processes. The function of the FLCN/FNIP complex is chiefly mediated through the regulation of two key protein kinases: the mTORC1 and AMPK (Moreno-Corona et al., 2023). The FLCN/FNIP complex promotes balance by coordinating mTORC1 and AMPK activities, with the crosstalk between these pathways further orchestrating metabolic adaptation to nutrient and energy conditions (Figure 6C) (Manford et al., 2020).

Iris E and colleagues demonstrated that disruption of FLC of human renal tubular epithelial cells (RPTEC/TERT1) activates TFE3, which upregulates the expression of its E-box targets, including Ras Related GTP Binding D (RRAGD) and GPNMB, without altering mTORC1 activity. Loss of the FLCN and FNIP1/FNIP2 complex triggered an interferon-independent response gene. FLCN deficiency promoted STAT2 recruitment to chromatin and decelerated cell proliferation, underscoring the FLCN and FNIP1/FNIP2 complex’s distinctive role in the development of human renal tumors and identifying potential prognostic biomarkers (Figure 6D) (Rallis et al., 2013; Schmidt and Linehan, 2018). Hisashi’s scientific team observed that in organs exhibiting a phenotype under Fnip1 deficiency, Fnip2 mRNA copy numbers are lower than those of Fnip1 but comparable to Fnip1 mRNA levels in mouse kidneys. Targeted dual inactivation of FNIP1/FNIP2 led to the enlargement of polycystic kidneys. Heterozygous FNIP1/homozygous FNIP2 double knockout mice developed renal cancer at 24 months, akin to heterozygous FLCN knockout mouse models, thereby reinforcing the significance of FNIP1 and Fnip2 in tumor suppression and suggesting potential molecular targets for novel renal cancer therapies (Hasumi et al., 2016; Mo et al., 2019). Professor Leeanna found that FLCN, FNIP1, and FNIP2 are downregulated in various human cancers, particularly in poorly prognostic invasive basal-like breast cancer, as opposed to luminal, less aggressive subtypes. In luminal breast cancer, the loss of FLCN activates TFE3, which then induces pathways promoting tumor growth, such as autophagy, lysosome biogenesis, aerobic glycolysis, and angiogenesis. Importantly, the induction of aerobic glycolysis and angiogenesis in FLCN-deficient cells results from the activation of the PGC-1α/HIF-1α pathway, which is TFE3-dependent, thereby directly linking TFE3 to Warburg metabolic reprogramming and angiogenesis. These findings underscore the broad role of the dysregulated FLCN/TFE3 tumor suppressor pathway in human cancers (de Martín Garrido and Aylett, 2020; Skidmore et al., 2022). Masaya’s team reaffirmed that mutations in FLCN and its interacting partner FNIP1, involved in Birt-Hogg-Dubé syndrome, may play a role in energy and nutrient sensing through the AMPK and mTOR signaling pathways. Laura S’s research identified that germline mutations on chromosome 17 in the FLCN gene are responsible for Birt-Hogg-Dubé syndrome, with BHD-associated renal tumors displaying inactivation of the wild-type FLCN allele due to somatic mutations or chromosomal loss, thus confirming FLCN as a tumor suppressor gene consistent with the classic two-hit model. FLCN interacts with two novel proteins, FNIP1 and FNIP2, and the negative regulator of mTOR, AMPK. Understanding the FLCN pathway may lead to the development of effective targeted systemic treatments for the disease (Figure 6E) (Furuya and Nakatani, 2013; Zhou et al., 2017).

Through its interaction with FLCN, FNIP1 modulates the mTOR signaling pathway, influencing cellular energy metabolism and viability (Kennedy et al., 2016). Dysfunctions in FNIP1 are associated with various types of cancer, including renal cell carcinoma (Woodford et al., 2021). In renal cancer, reduced expression of FNIP1 is correlated with increased tumor aggressiveness and malignancy (Clausen et al., 2020). Additionally, FNIP1 regulates cellular responses to hypoxic conditions, further linking these to metabolic reprogramming and resistance development in tumors (Schmidt, 2013). Single-cell sequencing technologies have been employed to explore variations in FNIP1 expression across different tumor cells, elucidating FNIP1’s complex role in tumor heterogeneity and progression (Tsun et al., 2013). These studies suggest that FNIP1 may have distinct functions and impacts across various tumor subtypes and microenvironments, offering new insights for precision medicine (Lawrence et al., 2019). Future research should delve into the specific mechanisms of FNIP1 in various cancers and investigate how these findings can be translated into clinical applications, potentially leading to more targeted and effective treatments.

## 8 FNIP1: a multidimensional regulator in mitochondrial, cardiovascular, and musculoskeletal diseases

FNIP1, a protein involved in various biological processes, particularly in regulating the mTOR signaling pathway and cellular energy metabolism, demonstrates significant potential for clinical applications. Research on FNIP1’s functions offers new insights and potential strategies for treating mitochondrial diseases, cardiovascular diseases, musculoskeletal disorders, disease prognosis assessment, and personalized medicine. FNIP1 plays a crucial role in regulating cellular energy metabolism and mitochondrial function, making it a potential biomarker and therapeutic target in the study and treatment of mitochondrial diseases. Studies indicate that FNIP1 may influence mitochondrial function and stability by regulating processes such as mitochondrial biogenesis, membrane potential, and oxidative phosphorylation (Lawrence et al., 2019). Consequently, therapeutic strategies targeting FNIP1 could represent a new approach to the treatment of mitochondrial diseases. Further research into the mechanisms of FNIP1’s role in the progression of mitochondrial diseases will aid in understanding the pathogenesis of these conditions and provide a theoretical basis for developing new treatment strategies. FNIP1 is clsely associated with the onset and progression of cardiovascular diseases. Initially, FNIP1 is involved in regulating cellular energy metabolism and glucose homeostasis, which are linked to common metabolic disorders in cardiovascular diseases such as atrial fibrillation and arrhythmias (Nakamura et al., 2021; Schmidt and Linehan, 2015). Studies indicate that metabolic disorders are a significant cause of cardiovascular diseases, and FNIP1 may act as a key regulator in these disorders, playing a crucial role in the pathogenesis of cardiovascular conditions (Hong et al., 2018; Zhang H. et al., 2023). Furthermore, FNIP1 is closely related to processes such as cardiomyocyte proliferation, apoptosis, and angiogenesis, which play key roles in the development of cardiovascular diseases. Research suggests that abnormal expression of FNIP1 may affect the function of endothelial cells, thereby influencing the stability of blood vessel walls and blood flow, ultimately impacting cardiovascular health (Hasumi et al., 2016; Woodford et al., 2021). Consequently, FNIP1 could serve as a biomarker for assessing cardiovascular disease risk and predicting disease progression. Moreover, studies have found that the expression levels of FNIP1 in patients with cardiovascular diseases are closely linked to treatment outcomes (De Conti et al., 2015; Ren et al., 2021), potentially making FNIP1 an important marker for evaluating the effectiveness of cardiovascular disease treatments and guiding personalized therapy.

FNIP1 also holds promising clinical application prospects in skeletal muscle diseases. It plays a critical role in regulating the metabolism, growth, and function of skeletal muscles, making it a potential biomarker and therapeutic target for the study and treatment of skeletal muscle diseases. Skeletal muscle diseases often involve muscle atrophy, degeneration, and dysfunction, and as a key regulator of cellular energy metabolism and protein synthesis, abnormal expression or dysfunction of FNIP1 can impair skeletal muscle function (Hasumi et al., 2008; Yin et al., 2022). Therefore, studying the expression levels of FNIP1 in patients with skeletal muscle diseases and their correlation with disease severity and prognosis can aid in assessing disease progression and predicting patient outcomes. Some skeletal muscle diseases’ pathogenesis involves mitochondrial dysfunction, and as a crucial regulator of mitochondrial function, abnormal expression or dysfunction of FNIP1 could lead to mitochondrial impairment, thereby affecting skeletal muscle function and stability (Parmar et al., 2022; Zhang et al., 2017). FNIP1 may be involved in regulating processes such as protein synthesis, cellular signaling, and muscle regeneration in skeletal muscles, affecting muscle growth and repair. Therefore, therapeutic strategies targeting FNIP1 could represent a new avenue for treating skeletal muscle diseases and provide a theoretical basis for developing new treatment approaches. The interaction between FNIP1 and FLCN critically regulates the mTOR pathway, affecting cellular responses to energy and nutrients, with aberrant activation of these pathways closely associated with the progression of mitochondrial diseases. Consequently, FNIP1 emerges as a potential pharmacological target for regulating these key biological processes. The development of drugs targeting FNIP1, whether small molecule inhibitors or activators, may pave new therapeutic pathways, particularly for types of mitochondrial diseases where traditional treatments are ineffective. Moreover, FNIP1 also shows broad potential in drug resistance and as a biomarker (Sun et al., 2021; Tsun et al., 2013). With the advancement of personalized medicine, the expression and functional status of FNIP1 could help physicians more accurately determine disease prognosis, select the most appropriate treatment options, and enhance therapeutic outcomes. Although FNIP1 has shown significant potential in laboratory research and preclinical models, its efficacy and safety in clinical applications still require validation through systematic clinical trials. Future research needs to address the dosage, pharmacodynamics, side effects, and long-term effects of FNIP1-targeted therapies. Additionally, research should also explore the role of FNIP1 in oncological diseases, such as its potential involvement in metabolic disorders, neurological diseases, and other possible pathological conditions. The mechanism of action of FNIP1 in clinical practice is shown in Table 3.

**TABLE 3:** Clinical and experimental evidence that FNIP1 modulates disease.

Clinical disease	Chemical compound	Model	Mechanism of action of FNIP1	Clinical therapeutic implications	References
Pancreatic cancer	MEF2A and MEF2D	Tumor cells	The FLCN-FNIP1 complex, functioning as an RRAGC-RRAGD GTPase, facilitates the recruitment and activation of mTORC1 at lysosomes	This study uncovered the transcriptional regulation of mTORC1, which drives cell anabolism and proliferation	Lawrence et al. (2019)
Kidney tumors	FLCN	FNIP2 knockout mice	FNIP1 and FNIP2 have inhibitory functions on FLCN tumors	Molecular targets for the development of new therapies for kidney cancer	Manford et al. (2021)
Bert-Hogg-Dolby syndrome	Cytoplasmic adaptor proteins	FNIP1 knockout mice	Deletion of Fnip1 synergizes with deletion of Tsc1, causing mTOR to overactivate, increase Erk activation, and accelerate the development of PKD.	The role of FNIP1 in the regulation of kidney development and function is defined	Shen et al. (2019)
Bert-Hogg-Dolby syndrome	Tumor suppressor proteins encoded by LKB1, TSC1/2, and PTEN	Tumor cells	FNIP1 overexpression enhances FLCN phosphorylation, indicating that FLCN may be regulated through mTOR and AMPK signaling pathways	FLCN and FNIP1 may be involved in energy and nutrient sensing through the AMPK and mTOR signaling pathways	Baba et al. (2006)
Bert-Hogg-Dolby syndrome	—	Breast cancer FLCN deficient cells	Aerobic glycolysis and angiogenesis in FLCN-deficient cells are regulated by the PGC-1α/HIF-1α pathway	Studies support the role of the dysregulated FLCN/TFE3 tumor suppressor pathway in human cancer	Fromm, Lawrence and Hurley (2020)
Bert-Hogg-Dolby syndrome	β-TRCP and CK1	Kidney cancer cells	Degradation of FNIP2 results in lysosomal dissociation of FLCNs and subsequent mTOR lysosomal binding	SCFβ-TRCP negatively regulates the FLCN complex by promoting FNIP degradation, providing insights into the pathogenesis of BHD-associated renal cancer	Skidmore et al. (2022)
Hepatocellular carcinoma	Whole genome and transcriptome sequencing	30 surgically resectable tumors	NR1H4 fusions involving gene chaperones EWSR1, GNPTAB, and FNIP1 were detected and validated in 2 non-cirrhosis samples	Genomic therapy may be appropriate for both subtypes of HCC for disease progression	Pan et al. (2012)
Kidney cancer	—	Tumor cells	AMPK-TSC1/2-mTOR and PI3K-Akt-mTOR interactions. TSC1-TSC2 is located downstream of AMPK and negatively regulates mTOR in response to cellular energy deficits	Mutations in kidney cancer genes, such as FLCN, disrupt metabolic pathways, highlighting kidney cancer as a disease of cellular metabolism	Baba et al. (2012)
Bert-Hogg-Dolby syndrome	—	Chromophilic and clear cell tumors in patients with BHD	Three novel phosphorylation sites (Ser406, Ser537, and Ser542) within FLCNs were identified that were induced by ULK1 overexpression	It reveals a new direction in the regulation of ULK1 autophagy and directly links it to autophagy through GABARAP and ULK1	Hasumi et al. (2008)
Cancer	mTORC1 kinase	Tumor cells	RagC/D interacts with GDP in order to have GAP activity on RagC/D	The role of RagC/D in mTORC1 activation and the molecular function of FLCN tumor suppressor genes were revealed	Tsun et al. (2013)
Being overly obese	—	Mice lacking adipocyte FNIP1	FNIP1 binds to and promotes its activity with SERCA, the primary Ca2+ pump responsible for cytosolic Ca2+ removal	FNIP1 acts as a negative regulator of thermogenesis in beige adipocytes, linking intracellular Ca2+ dynamics to adipocyte browning	Saettini et al. (2021)
Reduction stress disorders	CUL2FEM1B	Cell	CUL2FEM1B selectively recruit reduced FNIP1. BEX controls FNIP1 ubiquitination to prevent premature degradation of FNIP1 to protect cells from ROS accumulation	FEM1B mutations and BEX deletion cause similar syndromes, highlighting the need for tight regulation of zinc-dependent reductive stress to maintain homeostasis	(Zhang et al., 2012)
Reduction stress disorders	—	FNIP1(−/−) mice	Thymic iNKT development in Fnip1 (−/−) mice was stalled at stage 2 (NK1.1 (−)CD44 (+)), but the development of CD4, CD8, γδ T cells, and NK cell lineages proceeded normally	Fnip1 maintains metabolic homeostasis in response to metabolic stress and is essential for iNKT cell development	Ulaş et al. (2024)
Obesity	Palmitate	C2C12 myotube	Palmitate reduces Myh7 expression and insulin sensitivity, while increasing FLCN and FNIP1 expression, AMPK inactivation, and decreasing PGC-1α expression	DHM prevents palmitate-induced slow-twitch fiber reduction via the FLCN-FNIP1-AMPK pathway, improving insulin resistance in obese patients	Rallis et al. (2013)
Metabolic diseases	—	FNIP1 knockout mice	Genetic disruption of PGC1α rescued normal levels of the type I fiber markers MyH7 and myoglobin in Fnip1-null mice	FNIP1 controls the specification of skeletal muscle fiber types	Dunlop et al. (2014)
Normal human skeletal muscle	—	Primary skeletal muscle cells	AMPK exercise-induced phosphorylation of FNIP1 (S220) regulates mitochondrial electron transfer chain complex assembly, fuel utilization, and motor performance	FNIP1 is used as a multifunctional AMPK effector for mitochondrial adaptation	Tsun et al. (2013)
Patients with limb-girdle muscular dystrophy	Muscle-derived IGF2	Osteoclasts	Myofiber-specific FNIP1 deficiency promotes the nuclear translocation of transcription factor EB, activating Igf2 transcription at the conserved promoter binding site	A musculoskeletal crosstalk mechanism is elucidated, bridging the gap between muscle dysfunction and bone loss	Ramírez et al. (2019)
Fast-moving versus slow-shrinking muscle fibers	—	FNIP1 null mice	Cultured Fnip1-null myofibers have a higher oxidative capacity, and isolated Fnip1-null skeletal muscle is more resistant to postcontractile fatigue than WT skeletal muscle	Inhibition of Fnip1 was identified as having therapeutic potential in muscular dystrophy disease	Dunlop et al. (2014)
Myogenic cells	miRNA (miR-208b)	Myogenic cells	miR-208b promotes fast-to-slow fiber conversion and oxidative metabolism via FNIP1, activating AMPK/PGC-1α signaling and mitochondrial biogenesis by targeting FNIP1	miR-208b can mediate skeletal muscle development and homeostasis by specifically targeting TCF12 and FNIP1	Jellusova and Rickert (2016)

Note: FLCN, folliculin; FBP, Fatty acid-binding protein; FNIP1, Folliculin Interacting Protein 1; MEF2D, Myocyte-Specific Enhancer Factor 2D; LKB1, Liver kinase B1; β-TRCP, β-transducin repeat-containing protein; CK, casein kinase; IGF2, Insulin-like growth factor II.

## 9 Conclusion and perspectives

FNIP1 has emerged as a pivotal player in cellular metabolism and signaling pathways, making it a compelling candidate for therapeutic targeting. This review delves into the clinical applications of FNIP1, emphasizing its potential as a drug target. We analyze recent advancements in FNIP1 research, elucidating its role in disease modulation and therapeutic efficacy. The landscape of targeted therapeutics has evolved significantly, with the identification of novel molecular targets that offer promise for precision medicine. FNIP1, a key regulator within the mTOR signaling pathway, has garnered attention due to its involvement in metabolic processes and disease pathogenesis. This review aims to provide a comprehensive overview of FNIP1’s clinical applications, focusing on its relevance as a drug target. By synthesizing findings from recent high-impact studies, we elucidate the potential of FNIP1 in clinical interventions. FNIP1 functions as a crucial modulator of the mTORC1 pathway, integrating signals related to cellular energy status and nutrient availability. It interacts with FLCN, forming a complex that regulates AMPK and mTORC1 activity. Disruptions in FNIP1 expression or function have been linked to metabolic disorders, tumorigenesis, and other pathologies, highlighting its significance in maintaining cellular homeostasis.

Cancer Therapy: FNIP1’s involvement in mTOR signaling makes it a viable target for cancer treatment. Preclinical studies have shown that FNIP1 inhibitors can reduce tumor growth and enhance the efficacy of existing therapies. Metabolic Disorders: By regulating AMPK activity, FNIP1 influences glucose metabolism and lipid homeostasis. Therapeutic modulation of FNIP1 could offer novel treatments for metabolic diseases. Neurodegenerative Diseases: Emerging evidence suggests that FNIP1 plays a role in autophagy and cellular stress responses, providing a potential target for neurodegenerative conditions such as Parkinson’s and Alzheimer’s disease.

While FNIP1 presents a promising target, several challenges must be addressed to realize its clinical potential. These include: Specificity and Off-target Effects: Developing highly specific FNIP1 inhibitors to minimize off-target effects. Biomarker Development: Identifying biomarkers for patient stratification and treatment monitoring. Combinatorial Approaches: Exploring combination therapies that include FNIP1 targeting to enhance therapeutic outcomes. FNIP1 stands at the forefront of potential therapeutic targets due to its integral role in cellular signaling and metabolism. Continued research and clinical trials are essential to fully harness its potential in treating a wide array of diseases. By advancing our understanding of FNIP1’s mechanisms and interactions, we pave the way for novel, targeted therapeutic strategies that could revolutionize clinical practice. This article discusses the important historical progress and structural features of FNIP1 and introduces its potential links and mechanisms of action with mitochondrial diseases, cardiovascular diseases, and reductive stress diseases through complex mechanisms such as AMPK cellular energy metabolism, mTOR signaling pathway, modification of cellular autophagy, and reductive stress response. Some research conclusions on these mechanisms may be inconsistent, but this also illustrates the complexity of FNIP1’s mechanisms of action. These differing viewpoints are the most interesting parts of scientific research and provide a scientific basis for becoming new therapeutic targets. In summary, as a multifunctional protein, FNIP1 plays an increasingly important role in future biomedical research and therapeutic development. By further exploring its specific roles and mechanisms in various diseases, we can look forward to developing new therapeutic strategies, and offering more effective and safer treatment options for patients.

## Glossary

AID: Activation-induced cytidine deaminase
AKT1: AKT serine/threonine kinase 1
AMPK: Adenosine 5′-monophosphate (AMP)-activated protein kinase
AMBPA1: Autophagy and beclin 1 regulator 1 gene
AXIN: Axis Inhibition Protein 1
ATG13: Autophagy Related 13
ATG14L: Autophagy-related protein 14-like
ATG9: Autophagy-related protein 9
CAMKK2: Calcium/calmodulin-dependent protein kinase 2
CBM: Carbohydrate-binding modules
CBS1: Cystathionine beta-synthase 1
CHK2: Checkpoint kinase 2
CCL8: C-C motif chemokine ligand 8
CXCL13: C-X-C motif ligand 13
EC: Endothelial cell
EXO1: Exonuclease 1
ERRα: Estrogen Receptor alpha
FLCN: Folliculin
FBP: Fatty acid-binding protein
FNIP1: Folliculin Interacting Protein 1
GATOR1: GAP activity toward Rags 1
GAL9: Growth arrest-specific protein 9
GDP: Guanosine diphosphate
MO25: MO25 alpha/beta
LKB1: Liver kinase B1
mTOR: Mammalian target of rapamycin
OPTN: Optineurin
PI3K: Phosphatidylinositide 3-kinase
PGC1α: Peroxisome proliferators-activated receptor γ coactivator l alpha
RPKM: Reads Per Kilobase of exon model per Million mapped reads
STRAD: STE20-related kinase adaptor protein
TAK1: Transforming growth factor beta-activated kinase 1
TSC1: Tuberous Sclerosis Complex 1
TSC2: Tuberous Sclerosis Complex
TCF12: Transcription Factor 12
ULK1: Unc-51 like autophagy activating kinase 1
USP9X: Ubiquitin Specific Peptidase 9X-linked
RACK1: Receptor of activated protein kinase C1
VPS34: Vacuolar Protein Sorting 34
