# Supplementary figures and images for: Action and therapeutic targets of folliculin interacting protein 1: a novel signaling mechanism in redox regulation
Source: Front Cell Dev Biol. 2025 Mar 12;13:1523489. doi: 10.3389/fcell.2025.1523489 (PMC11936992; doi:10.3389/fcell.2025.1523489)

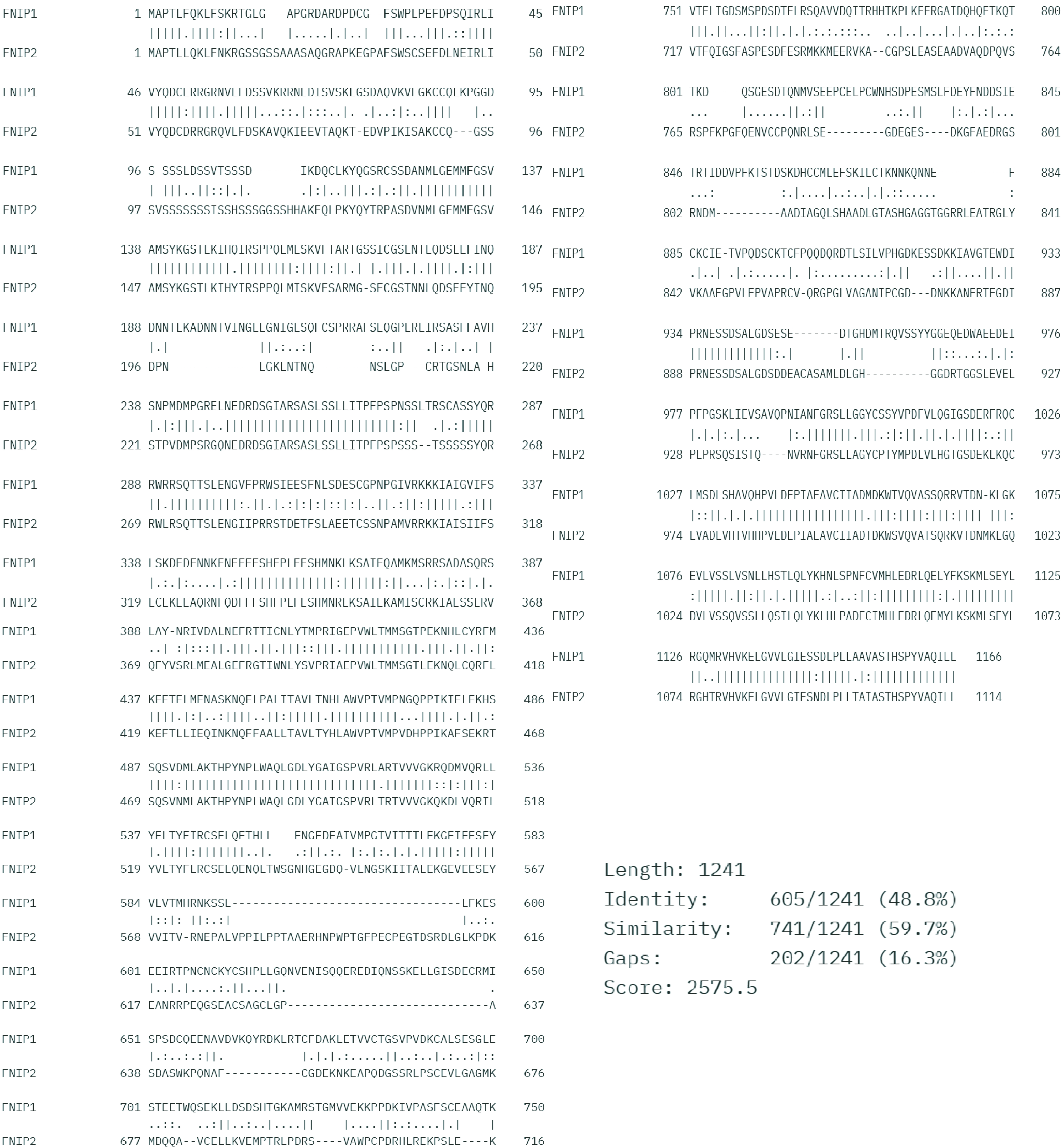

Supplement: Supplementary file 1 [file Image1.JPEG]
